# Associations between Yogurt, Dairy, Calcium, and Vitamin D Intake and Obesity among U.S. Children Aged 8–18 Years: NHANES, 2005–2008

**DOI:** 10.3390/nu7031577

**Published:** 2015-03-03

**Authors:** Debra R. Keast, Kathleen M. Hill Gallant, Ann M. Albertson, Carolyn K. Gugger, Norton M. Holschuh

**Affiliations:** 1 Food & Nutrition Database Research Inc., Okemos, MI 48864, USA; E-Mail: keastdeb@comcast.net; 2 Department of Nutrition Science, Purdue University, West Lafayette, IN 47907, USA; 3 Bell Institute of Health and Nutrition, General Mills, Inc., Minneapolis, MN 55427, USA; E-Mails: ann.albertson@genmills.com (A.M.A); carolyn.gugger@genmills.com (C.K.G.); 4 Statistics Department, General Mills, Inc., Minneapolis, MN 55440, USA; E-Mail: nort.holschuh@genmills.com

**Keywords:** yogurt, dairy, calcium, vitamin D, obesity, children

## Abstract

The aim of this study was to investigate associations of yogurt and dairy consumption with energy, macronutrient, calcium, and vitamin D intakes, and associations with indicators of overweight/obesity in U.S. children in the National Health and Nutrition Examination Survey (NHANES 2005–2008). Using 24-hour recall data, children 8–18 years of age were classified to dairy consumption groups of <1, 1 to <2, or 2+ dairy servings, and yogurt consumers were those who reported eating yogurt during at least one of two dietary intake interviews. NHANES anthropometric measurements were used, and BMI and BMI-for-age percentiles were calculated. Yogurt and dairy consumption were associated with higher intakes of calcium, vitamin D and protein. Yogurt intake was associated with lower total fat and saturated fat intakes and body fat as measured by subscapular skinfold thickness. This study supports consumption of yogurt and higher amounts of dairy as eating patterns associated with greater intake of specific shortfall nutrients, and lower body fat in U.S. children.

## 1. Introduction

The rate of obesity among children in the U.S. continues to be a major public health concern due to increased risk for health problems such as cardiovascular disease and type 2 diabetes [1,2]. Despite the high rate of obesity, a high proportion of U.S. children do not meet recommended intakes of essential micronutrients, including calcium and vitamin D [3,4]. These are particularly important nutrients during this stage, as adolescence is a period when bone mineral accrual rate is highest, and approximately 95% of adult peak bone mass is acquired by the age of twenty [5]. The 2010 Institute of Medicine Dietary Reference Intake committee set the Recommended Dietary Allowances at 1300 mg/day calcium and 600 IU/day vitamin D for children ages 9–18 years to achieve bone health [6], and the 2010 Dietary Guidelines for Americans recommended three servings per day of low or no-fat dairy [7]. Yet, most U.S. children do not meet these recommendations [3,8,9]. Additionally, it has been shown that the nutrients from dairy foods, including calcium and vitamin D, are difficult to replace with other foods in American diets [10]. Given the high rate of childhood obesity and its association with risk of morbidity and mortality, it is important to understand the associations of dairy foods and their constituent nutrients with obesity in children if public health messages urge increased consumption of these foods.

A recent review [11] summarizes results of current cross-sectional or longitudinal studies and randomized-controlled trials investigating the relationship between obesity and dairy intake by children. Several cross-sectional studies have shown an inverse relationship between body mass index (BMI) or adiposity and dairy intake in children [12,13,14,15], but this has not been consistent across all studies [16]. Additionally, randomized controlled trials of dairy interventions have been largely neutral [17,18,19,20,21,22]. Recent studies have also suggested the relationship between dairy foods and weight measures may be influenced by the form and matrix of the food [23,24]. Yogurt is distinct from other common fresh dairy products as it is a by-product of milk fermentation, and contains live and active bacteria [23]. The manufacturing and fermentation process also results in a more concentrated dairy product, as plain yogurt is higher in specific nutrients including calcium, potassium, and protein compared to milk [24]. Yogurt has been identified as a nutrient-dense food that may help Americans meet the recommended intake for dairy from the dietary guidelines [25], and cross-sectional studies have shown that yogurt intake is associated with better diet quality [26]. Observational and experimental studies on the effects of yogurt on obesity and weight maintenance have been mixed, with few studies focused specifically on children [25]. The purpose of the present study was to investigate associations of dairy and yogurt consumption with calcium, vitamin D, energy, and other micro- and macronutrient intakes, as well as the associations of dairy, yogurt, vitamin D and calcium intakes with indicators of overweight/obesity of U.S. children, utilizing a large, representative sample from the National Health and Nutrition Examination Survey (NHANES 2005–2008). We hypothesized that greater dairy and yogurt consumption is associated with greater intakes of calcium and vitamin D, but not associated with greater adiposity or obesity/overweight.

## 2. Experimental Section

### 2.1. Study Population

The continuous National Health and Nutrition Examination Survey (NHANES) is a cross-sectional survey that provides nationally representative data pertaining to the nutrition and health status of the U.S. population by selecting sample persons using a complex, multi-stage probability sampling design. Data collected from participants of the 2005–2006 and 2007–2008 NHANES were combined for these analyses [27]. The analytic sample included children aged 8–18 years and excluded pregnant or lactating females. Due to the fact that NHANES has stringent consent protocols and procedures which comply with federal laws to ensure confidentiality of information collected from individual participants [28], and the present study is a secondary analyses of data that lack personal identifiers, this study was exempted by the Institutional Review Board of Purdue University.

### 2.2. Dietary Intake Assessment

The What We Eat in America (WWEIA) dietary component of NHANES, conducted by the United States Department of Agriculture (USDA) Food Surveys Research Group (FSRG), includes two, non-consecutive 24-hour recall dietary intake interviews administered using an automated multiple-pass method [29,30]. The Day 1 24-hour dietary recall was conducted via in-person interview at the Mobile Examination Center, and the Day 2 24-hour recall was conducted via telephone interview. Survey participants 12 years and older completed the dietary interview on their own, proxy-assisted interviews were conducted with children 6–11 years of age. A detailed synopsis of the dietary interview methods has been described previously [31]. Only dietary interview data identified as complete and reliable as assessed by USDA FSRG staff were included in these analyses.

The USDA FSRG determined nutrient intake from foods reported in the 2005–2006 and 2007–2008 WWEIA/NHANES using their Food and Nutrient Database for Dietary Studies, versions 3.0, and 4.1, respectively [32,33]. MyPyramid food group intake from 2005 to 2006 and 2007 to 2008 WWEIA/NHANES foods that were also reported in 2003 to 2004 was determined using the MyPyramid Equivalent Database, version 2.0 (MPED 2.0) [34]. MyPyramid food group intake from foods that were unique to the 2005–2006 and 2007–2008 WWEIA/NHANES were determined using the Center for Nutrition Policy and Promotion Addendum to the MyPyramid Equivalents Database 2.0 [35]. The MPED 2.0 database defines total dairy consumption based on the reported intake of milk, cheese, yogurt and whey.

Participants were classified to dairy and yogurt consumption groups using 24-hour recall data. The MyPyramid Equivalents Database quantified dairy consumption as cup-equivalent servings per day, which will be referred to as “servings”. Classification of participants to groups consuming less than one serving (<1), one but less than 2 servings (1 to <2), or two or more (2+) dairy servings was based on MyPyramid dairy intake from the Day 1 24-hour recall. Yogurt consumers were defined as those who reported eating yogurt during one or both 24-hour dietary intake interviews in order to obtain a sample size sufficient to produce reliable estimates. Total daily energy, macronutrient, sodium, potassium, calcium, vitamin D intake was assessed using the Day 1 dietary recall for both dairy and yogurt consumption groups. Tertiles of calcium and vitamin D intake were determined for gender strata, and these cut-points were used to form groups.

### 2.3. Anthropometric Assessment

Waist circumference, skinfold thickness measurements (triceps and subscapular), weight and height were measured by trained personnel in a Mobile Examination Center (MEC) according to NHANES protocols [36]. Body mass index (BMI) was calculated as body weight (kg) divided by height (m) squared. The percentile of BMI-for-age was calculated using the Statistical Analysis Software program (SAS; Cary, NC, USA) for Centers for Disease Control and Prevention (CDC) Growth Charts (Centers for Disease Control and Prevention, Atlanta, GA, USA). Children who had a BMI ≥85th percentile of BMI-for-age were classified as overweight/obese. Reference percentiles of waist circumference for children grouped by gender and year of age [37] were used to determine abdominal obesity defined as a waist circumference ≥85th percentile.

### 2.4. Statistical Analysis

The analytic dataset was prepared using the Statistical Package for Social Sciences (SPSS) Release 17.0.2 (IBM, Armonk, NY, USA) and all statistical analyses were performed using a standalone version of SUDAAN Release 10.0.1 (Research Triangle Institute, Research Triangle Park, NC, USA) to adjust the variance for the clustered sample design. The least square mean and standard error (SE) were determined from sample-weighted data using the generalized multiple regression procedure (PROC REGRESS) to discern between-group differences in the mean level of the dependent variable, after adjusting for potential confounders. The covariates used in analyses of nutrient intake were energy (kcal) intake, gender, years of age, and race-ethnicity. Gender, years of age, and race-ethnicity were covariates in analyses of energy (kcal) intake and energy intake from macronutrients expressed as a percentage (%) of daily energy intake. In analyses of anthropometric dependent variables, model 1 covariates included energy (kcal) intake, gender, years of age, and race-ethnicity; and model 2 covariates included energy (kcal) intake, gender, years of age, race-ethnicity, poverty income level, physical activity level, TV/video/computer use, alcohol use, and tobacco use. The adjusted prevalence of a dichotomous variable such as obesity was determined by calculating the least-square mean of that variable. Data are presented as mean ± SE, and statistical significance was set at *p* < 0.01 and *p* < 0.05 levels using the Bonferroni method to adjust for multiple comparisons.

## 3. Results 

### 3.1. Energy and Nutrient Intake

Of the 3821 NHANES 2005–2008 participants aged 8–18 years who completed both 24-hour recall interviews, 35 females who were either pregnant (*n* = 29) or lactating (*n* = 6) were excluded, leaving a final analytical sample of 3786 (2005–2006: 2309, 2007–2008: 1477). Based on Day 1 and Day 2 dietary recalls, 8.5% of participants were yogurt consumers, with the majority (91.5%) of children not consuming yogurt. Approximately one-third (30%) of children reported consuming less than one serving of dairy, 28% reported consuming one, but less than 2 servings, and 41% of children consumed 2 or more servings of dairy each day. Differences in covariate-adjusted mean energy, macronutrient, calcium, vitamin D, sodium, and potassium intakes are shown in Table 1 and Table 2, respectively. Yogurt consumers had lower total fat (% daily energy) and saturated fat (g/day, % daily energy) intakes than non-yogurt consumers, while energy (kcal/day), carbohydrate (g/day), total sugars (g/day), added sugars (g/day), and total fat (g/day) did not differ between groups (*p* < 0.05) (Table 1). Yogurt consumers also had higher intakes of calcium, vitamin D, protein, and potassium than non-yogurt consumers (*p* < 0.01). Greater dairy consumption was associated with higher intakes of energy (kcal/day), protein (g/day) and saturated fat (g/day, % daily energy) and lower intakes of carbohydrate (g/day) and added sugars (g/day) (*p* < 0.05) (Table 2). Higher dairy consumption was also associated with higher calcium, vitamin D, and potassium intakes (*p* < 0.05).

**Table 1:** Covariate-adjusted mean energy, macronutrient, calcium, vitamin D, sodium, and potassium intake by yogurt consumption groups ^1^.

Nutrient ^2^	Non-Yogurt Consumer	Yogurt Consumer ^3^
	(*n* = 3506)	(*n* = 280)
	Mean ± SE	Mean ± SE
**Energy** (kcal)	2133 ± 23	2081 ± 58
**Protein**
(g)	75.2 ± 0.6	79.6 ± 1.5 **
(% energy)	14.2 ± 0.1	15.1 ± 0.3 **
**Total Fat**
(g)	80.3 ± 0.7	76.8 ± 1.8
(% energy)	33.5 ± 0.3	31.6 ± 0.7 *
**Saturated Fat**
(g)	28.2 ± 0.3	26.4 ± 0.8 *
(% energy)	11.7 ± 0.1	10.9 ± 0.3 *
**Carbohydrate**
(g)	280.8 ± 1.7	285.4 ± 4.4
(% energy)	53.3 ± 0.3	54.4 ± 0.8
**Total Sugars**
(g)	138.4 ± 1.8	143.0 ± 4.7
(% energy)	26.3 ± 0.3	27.3 ± 0.9
**Added Sugars**
(g)	94.7 ± 1.7	92.0 ± 4.3
(% energy)	17.6 ± 0.3	17.3 ± 0.7
**Micronutrients**		
Calcium (mg)	1001 ± 14	1105 ± 27 **
Vitamin D (μg) ^4^	4.97 ± 0.12	5.97 ± 0.33 **
Sodium (mg)	3383 ± 43	3334 ± 87
Potassium (mg)	2215 ± 27	2478 ± 63 **

^1^ Sample of children includes NHANES, 2005–2008, participants aged 8–18 years who completed two dietary recalls, and excludes pregnant or lactating females; ^2^ Covariates in analyses of energy (kcal) and macronutrient (% energy) intake include gender, years of age and race-ethnicity; covariates in analyses of macronutrient (gram) and micronutrient intake include energy (kcal) intake, gender, years of age and race-ethnicity; ^3^ Yogurt consumers defined by consumption of yogurt on one or both 24-hour recalls; ^4^ Vitamin D conversion: 1 μg = 40 IU; ** *p* < 0.01, * *p* < 0.05 significant difference between yogurt consumer and non-yogurt consumer.

**Table 2:** Covariate-adjusted mean energy, macronutrient, calcium, vitamin D, sodium, potassium intake by dairy consumption groups ^1^.

Nutrient ^3^	Low Dairy ^2^	Middle Dairy	High Dairy
	<1 Serving	1 to <2 Servings	2+ Servings
	(*n* = 1239)	(*n* = 1098)	(*n* = 1449)
	Mean ± SE	Mean ± SE	Mean ± SE
**Energy** (kcal)	1747 ± 25 ^a^	2006 ± 33 ^b^	2496 ± 30 ^c^
**Protein**
(g)	69.4 ± 0.8 ^a^	75.3 ± 1.2 ^b^	80.3 ± 0.8 ^c^
(% energy)	13.2 ± 0.2 ^a^	14.5 ± 0.2 ^b^	15.0 ± 0.2 ^b^
**Total Fat**
(g)	80.7 ± 1.1	79.8 ± 0.9	79.7 ± 0.8
(% energy)	32.8 ± 0.4	33.1 ± 0.4	33.8 ± 0.3
**Saturated Fat**
(g)	25.2 ± 0.6 ^a^	27.1 ± 0.4 ^b^	30.8 ± 0.4 ^c^
(% energy)	10.2 ± 0.2 ^a^	11.4 ± 0.1 ^b^	12.9 ± 0.1 ^c^
**Carbohydrate**
(g)	286.2 ± 2.7 ^a^	281.8 ± 2.3 ^a,b^	277.1 ± 1.9 ^b^
(% energy)	55.1 ± 0.5 ^a^	53.4 ± 0.4 ^b^	52.1 ± 0.4 ^b^
**Total Sugars**
(g)	138.9 ± 2.9	136.0 ± 3.1	140.5 ± 2.1
(% energy)	26.8 ± 0.5	25.8 ± 0.5	26.4 ± 0.4
**Added Sugars**
(g)	104.8 ± 3.0 ^a^	95.9 ± 2.7 ^a^	85.9 ± 2.5 ^b^
(% energy)	19.7 ± 0.5 ^a^	17.3 ± 0.4 ^b^	16.2 ± 0.5 ^b^
**Micronutrients**			
Calcium (mg)	635 ± 8 ^a^	874 ± 17 ^b^	1381 ± 17 ^c^
Vitamin D (μg) ^4^	2.11 ± 0.13 ^a^	4.13 ± 0.11 ^b^	7.88 ± 0.2 ^c^
Sodium (mg)	3331 ± 34	3509 ± 96	3324 ± 49
Potassium (mg)	2027 ± 36 ^a^	2175 ± 32 ^b^	2437 ± 45 ^c^

^1^ Sample of children includes NHANES, 2005–2008, participants aged 8–18 years who completed two dietary recalls, and excludes pregnant or lactating females; ^2^ Dairy consumption levels defined by cup-equivalent servings of dairy consumed on the first 24-hour recall; ^3^ Covariates in analyses of energy (kcal) and macronutrient (% energy) intake include gender, years of age; ^4^ Vitamin D conversion: 1 μg = 40 IU; ^a,b,c^ Paired groups are significantly different (*p* < 0.05, with Bonferronni adjustment) if notations do not share the same alphabetic character.

### 3.2. Anthropometric Variables

Covariate-adjusted differences in means in obesity/adiposity measures between yogurt consumers and non-yogurt consumers, and between dairy consumption groups are shown in Table 3 and Table 4, respectively. Yogurt consumers had lower prevalence of overweight or obesity, lower BMI-for-age, lower waist circumference, and smaller subscapular skinfold after adjusting for demographic and energy intake differences than non-yogurt consumers in Model 1. However, only differences in BMI-for-age, waist circumference, and subscapular skinfold persisted in the fully-adjusted Model 2 (*p* < 0.05) (Table 3).

Subscapular skinfold thickness was the only indicator of obesity or adiposity that was inversely associated with dairy consumption (Table 4). In both Model 1 and Model 2, high dairy (2+ servings) consumers had smaller subscapular skinfold than low dairy (<1 serving) consumers (*p* < 0.05) (Table 4). Subscapular skinfold was lower for yogurt consumers than non-yogurt consumers, and for consumers of 2+ *vs.* < 1 dairy servings in Model 2, and yogurt consumers also had lower waist circumference than non-yogurt consumers (*p* < 0.05) (Table 4).

**Table 3:** Covariate-adjusted mean value of obesity or adiposity indicator by yogurt consumption groups ^1^.

Indicator of Obesity or Adiposity ^3^	Non-Yogurt Consumer	Yogurt Consumer ^2^
	(*n* = 3506)	(*n* = 280)
	Mean ± SE	Mean ± SE
**Anthropometric Indicators**		
**Weight (kg)**		
Model 1	56.1 ± 0.6	54.2 ± 0.9
Model 2	56.1 ± 0.5	54.4 ± 0.9
**Waist circumference (cm)**		
Model 1	76.9 ± 0.5	74.5 ± 1.0 *
Model 2	77.0 ± 0.5	74.5 ± 1.0 *
**Abdominal adiposity prevalence (%)**		
Model 1	20.6 ± 1.6	15.3 ± 2.9
Model 2	20.6 ± 1.5	15.8 ± 3.3
**Triceps skinfold (mm)**		
Model 1	15.5 ± 0.3	14.4 ± 0.7
Model 2	15.5 ± 0.2	14.5 ± 0.7
**Subscapular skinfold (mm)**		
Model 1	12.9 ± 0.3	11.1 ± 0.5 *
Model 2	12.9 ± 0.3	11.3 ± 0.6 *
**Calculated Indicators**		
**Body Mass Index (kg/m^2^)**		
Model 1	22.0 ± 0.2	21.3 ± 0.3 *
Model 2	22.0 ± 0.2	21.3 ± 0.3 *
**Percentile of BMI-for-age (%)**		
Model 1	64.8 ± 1.2	60.4 ± 2.9
Model 2	64.7 ± 1.2	61.3 ± 2.7
**Overweight/obesity prevalence (%)**		
Model 1	36.2 ± 1.9	27.0 ± 4.0 *
Model 2	35.7 ± 1.8	27.6 ± 4.6

^1^ Sample of children includes NHANES, 2005–2008; participants aged 8–18 years who completed two dietary recalls, and excludes pregnant or lactating females; ^2^ Yogurt consumers defined by consumption of yogurt on one or both 24-hour recalls; ^3^ Model 1 covariates include energy (kcal) intake, gender, years of age, race-ethnicity; and alcohol use (days/year), and tobacco use in last 5 days (yes/no) and Model 2 covariates include energy (kcal) intake, gender, years of age, race-ethnicity, poverty income level, physical activity level, TV/video/computer use (h/day), alcohol use (days/year), and tobacco use in last 5 days (yes/no); * *p* < 0.05 significant difference between yogurt consumer and non-yogurt consumer.

**Table 4:** Covariate-adjusted mean value of obesity or adiposity indicator by dairy consumption groups ^1^.

Indicator of Obesity or Adiposity ^3^	Low Dairy ^2^	Middle Dairy	High Dairy
	<1 Serving	1 to <2 Servings	2+ Servings
	(*n* = 1239)	(*n* = 1098)	(*n* = 1449)
	Mean ± SE	Mean ± SE	Mean ± SE
**Anthropometric Indicators**			
**Weight (kg)**			
Model 1	55.8 ± 0.8	56.1 ± 1.2	55.9 ± 0.8
Model 2	55.5 ± 0.8	56.5 ± 1.1	56.0 ± 0.8
**Waist circumference (cm)**			
Model 1	77.5 ± 0.6	76.8 ± 1.1	76.1 ± 0.6
Model 2	77.4 ± 0.7	76.9 ± 1.1	76.2 ± 0.6
**Abdominal adiposity prevalence (%)**			
Model 1	22.7 ± 2.1	19.2 ± 3.0	19.1 ± 1.8
Model 2	22.4 ± 2.2	19.3 ± 2.9	19.3 ± 1.7
**Triceps skinfold (mm)**			
Model 1	16.0 ± 0.4	15.3 ± 0.5	15.2 ± 0.3
Model 2	15.9 ± 0.4	15.3 ± 0.5	15.2 ± 0.3
**Subscapular skinfold (mm)**			
Model 1	13.6 ± 0.3 ^a^	12.6 ± 0.5 ^a,b^	12.1 ± 0.3 ^b^
Model 2	13.5 ± 0.3 ^a^	12.6 ± 0.5 ^a,b^	12.2 ± 0.3 ^b^
**Calculated Indicators**			
**Body Mass Index (kg/m^2^)**			
Model 1	22.2 ± 0.3	21.9 ± 0.4	21.8 ± 0.2
Model 2	22.1 ± 0.3	22.0 ± 0.4	21.9 ± 0.2
**Percentile of BMI-for-age (%)**			
Model 1	65.8 ± 1.7	62.6 ± 2.0	64.6 ± 1.3
Model 2	65.1 ± 1.8	63.0 ± 1.9	65.0 ± 1.3
**Overweight/obesity prevalence (%)**			
Model 1	39.1 ± 1.9	33.4 ± 3.5	34.0 ± 1.8
Model 2	37.7 ± 1.8	33.8 ± 3.4	33.9 ± 1.8

^1^ Sample of children includes NHANES, 2005–2008, participants aged 8–18 years who completed two dietary recalls, and excludes pregnant or lactating females; ^2^ Dairy consumption levels defined by cup-equivalent servings of dairy consumed on the first 24-hour recall; ^3^ Model 1 covariates include energy (kcal) intake, gender, years of age, race-ethnicity; and Model 2 covariates include energy (kcal) intake, gender, years of age, race-ethnicity, poverty income level, physical activity level, TV/video/computer use (h/day), alcohol use (days/year), and tobacco use in last 5 days (yes/no); ^a,b^ Paired groups are significantly different (*p* < 0.05, with Bonferronni adjustment) if notations do not share the same alphabetic character.

Covariate-adjusted differences in mean obesity/adiposity measures between calcium intake tertile groups and differences between vitamin D intake tertile groups are shown in Table 5 and Table 6, respectively. Subscapular skinfold thickness was the only indicator of obesity or adiposity that was associated with calcium and vitamin D intake. After adjusting for gender, race-ethnicity, years of age and energy intake (Model 1), and also after physical activity and other covariates were added to the model (Model 2), the high calcium and vitamin D intake tertile group had smaller subscapular skinfold than the low calcium and vitamin D intake tertile group (*p* < 0.05) (Table 5 and Table 6). In both Model 1 and Model 2, the high vitamin D intake tertile group had a lower prevalence of overweight or obesity compared to the low vitamin D intake tertile group (*p* < 0.05) (Table 6). Intakes of yogurt and dairy high in calcium and vitamin D intake were associated with a lower prevalence of overweight or obesity.

**Table 5:** Covariate-adjusted mean value of obesity or adiposity indicator by calcium intake ^1^.

Indicator of Obesity or Adiposity ^3^	Calcium Intake ^2^ Tertile Group		
	Low	Middle	High
	(*n* = 1356)	(*n* = 1274)	(*n* = 1156)
	Mean ± SE	Mean ± SE	Mean ± SE
**Anthropometric Indicators**			
**Weight (kg)**			
Model 1	55.8 ± 0.7	55.8 ± 1.2	56.2 ± 0.9
Model 2	55.9 ± 0.7	55.9 ± 1.1	56.2 ± 0.8
**Waist circumference (cm)**			
Model 1	77.5 ± 0.5	76.5 ± 1.0	76.1 ± 0.7
Model 2	77.5 ± 0.6	76.6 ± 1.0	76.2 ± 0.7
**Abdominal adiposity prevalence (%)**			
Model 1	22.0 ± 1.6	19.1 ± 2.6	19.6 ± 2.2
Model 2	21.7 ± 1.8	19.2 ± 2.6	19.8 ± 1.9
**Triceps skinfold (mm)**			
Model 1	15.9 ± 0.3	15.3 ± 0.5	15.1 ± 0.3
Model 2	15.8 ± 0.3	15.3 ± 0.4	15.2 ± 0.3
**Subscapular skinfold (mm)**			
Model 1	13.5 ± 0.3 ^a^	12.6 ± .5 ^a,b^	12.1 ± 0.3 ^b^
Model 2	13.4 ± 0.3 ^a^	12.6 ± 0.4 ^a,b^	12.2 ± 0.3 ^b^
**Calculated Indicators**			
**Body Mass Index (kg/m^2^)**			
Model 1	22.1 ± 0.2	21.9 ± 0.4	21.9 ± 0.3
Model 2	22.1 ± 0.2	21.9 ± 0.4	21.9 ± 0.3
**Percentile of BMI-for-age (%)**			
Model 1	65.0 ± 1.6	63.6 ± 1.9	64.7 ± 1.5
Model 2	64.7 ± 1.6	63.5 ± 1.8	65.1 ± 1.4
**Overweight/obesity prevalence (%)**			
Model 1	37.7 ± 1.8	34.6 ± 3.2	33.8 ± 2.2
Model 2	37.3 ± 1.8	34.3 ± 2.9	33.5 ± 2.3

^1^ Sample of children includes NHANES, 2005–2008, participants aged 8–18 years who completed two dietary recalls, and excludes pregnant or lactating females; ^2^ Calcium intake levels defined by tertiles of calcium intake reported on the first 24-hour recall by gender groups; ^3^ Model 1 covariates include energy (kcal) intake, gender, years of age, race-ethnicity; and Model 2 covariates include energy (kcal) intake, gender, years of age, race-ethnicity, poverty income level, physical activity level, TV/video/computer use (h/day), alcohol use (days/year), and tobacco use in last 5 days (yes/no); ^a,b^ Paired groups are significantly different (*p* < 0.05, with Bonferronni adjustment) if notations do not share the same alphabetic character.

**Table 6:** Covariate-adjusted mean value of obesity or adiposity indicator by vitamin D intake ^1^.

Indicator of Obesity or Adiposity ^3^	Vitamin D Intake ^2^ Tertile Group		
	Mean ± SE	Mean ± SE	Mean ± SE
	Low	Middle	High
	(*n* = 1370)	(*n* = 1246)	(*n* = 1170)
**Anthropometric Indicators**			
**Weight (kg)**			
Model 1	56.5 ± 1.2	55.2 ± 0.8	56.1 ± 0.8
Model 2	56.4 ± 1.2	55.5 ± 0.7	56.1 ± 0.8
**Waist circumference (cm)**			
Model 1	77.8 ± 0.9	76.5 ± 0.9	75.7 ± 0.7
Model 2	77.8 ± 0.9	76.7 ± 0.8	75.7 ± 0.7
**Abdominal adiposity prevalence (%)**			
Model 1	22.7 ± 2.4	20.1 ± 2.5	17.7 ± 1.7
Model 2	22.4 ± 2.3	20.4 ± 2.4	17.8 ± 1.4
**Triceps skinfold (mm)**			
Model 1	16.0 ± 0.3	15.4 ± 0.3	14.9 ± 0.3
Model 2	15.9 ± 0.3	15.4 ± 0.3	15.0 ± 0.3
**Subscapular skinfold (mm)**			
Model 1	13.5 ± 0.3 ^a^	12.6 ± 0.4 ^a^^,b^	12.1 ± 0.3 ^b^
Model 2	13.3 ± 0.3 ^a^	12.7 ± 0.4 ^a,b^	12.1 ± 0.3 ^b^
**Calculated Indicators**			
**Body Mass Index (kg/m^2^)**			
Model 1	22.3 ± 0.3	21.9 ± 0.3	21.7 ± 0.3
Model 2	22.2 ± 0.3	21.9 ± 0.3	21.7 ± 0.2
**Percentile of BMI-for-age (%)**			
Model 1	66.6 ± 1.6	63.9 ± 1.9	62.7 ± 1.7
Model 2	66.1 ± 1.5	64.2 ± 1.8	62.9 ± 1.6
**Overweight/obesity prevalence (%)**			
Model 1	40.9 ± 2.1 ^a^	32.8 ± 3.2 ^a,b^	32.4 ± 2.5 ^b^
Model 2	39.8 ± 1.9 ^a^	33.2 ± 3.2 ^a,b^	32.2 ± 2.5 ^b^

^1^ Sample of children includes NHANES, 2005–2008, participants aged 8–18 years who completed two recalls, and excludes pregnant or lactating females; ^2^ Vitamin D intake levels defined by tertiles of vitamin D intake on the first 24-hour recall by gender groups; ^3^ Model 1 covariates include energy (kcal) intake, gender, years of age, race-ethnicity; and Model 2 covariates include energy (kcal) intake, gender, years of age, race-ethnicity, poverty income level, physical activity level, TV/video/computer use (h/day), alcohol use (days/year), and tobacco use in last 5 days (yes/no); ^a,b^ Paired groups are significantly different (*p* < 0.05, with Bonferronni adjustment) if notations do not share the same alphabetic character.

## 4. Discussion

This evaluation of data from NHANES, 2005–2008, shows that yogurt and dairy consumption are associated with greater calcium and vitamin D intakes, and that higher yogurt, dairy, calcium, and vitamin D intake are associated with lower measures of adiposity in U.S. children. Both yogurt and dairy consumption were associated with more favorable nutrient intakes of calcium, vitamin D, protein, and potassium—nutrients for which yogurt and dairy are rich sources. Presumably, intakes of these nutrients are positively associated with yogurt and dairy intakes because these foods are the common nutrient sources, although concurrent dietary habits of yogurt and dairy consumers may also contribute to the positive associations between consumption of these foods and intake of constituent nutrients [10]. Greater dairy consumption was associated with higher energy intake as well as higher saturated fat intake (as % of daily energy). Conversely, yogurt consumption was not associated with higher energy intake and was actually associated with lower total and saturated fat intake (as % daily energy). An explanation for this discrepancy may be the wide range of fat contained in dairy products such as cheese, ice cream, and fluid milk, as well as higher fat foods that include dairy (e.g., cheese pizza). Fluid milk and cheese are the most commonly consumed dairy foods [7]. The majority of current fluid milk intake is from 2 percent milk or whole milk, with smaller amounts of low-fat (*i.e.*, 1 percent milk fat) or fat-free milk, and little cheese intake is in the lower fat form [7]. Comparatively, low-fat and fat-free yogurts represent a large proportion of the yogurt available and consumed in the United States. Low-fat and fat-free yogurts may be displacing other higher fat foods in the diet, or yogurt consumption may simply be a marker for a more healthful dietary pattern. A similar explanation for the association found between higher dairy consumption and lower added sugar consumption could be that lower sugar dairy foods (*i.e.*, fluid white milk) might displace higher sugar foods (*i.e.*, regular soda), or dairy consumption may be associated with other dietary choices that lead to lower added sugar intakes. However, these proposed dietary and nutrient intake relationships could not be established from this analysis.

Yogurt consumption was associated with lower BMI, as well as lower waist circumference and smaller subscapular skinfold thickness. Results from the multi-country European Healthy Lifestyle in Europe by Nutrition in Adolescence (HELENA) study also reported an inverse association between higher consumption of yogurt, milk, and yogurt-based beverages with waist circumferences and subscapular skinfold measures in girls, but not boys [38]. A recent study of yogurt consumption in the Framingham Offspring and Third Generation Cohorts found that adults who consumed yogurt (>0 times per week) had lower BMI in addition to other metabolic benefits [26]. Although we adjusted for potential confounders indicative of overall lifestyle healthfulness including total energy intake, physical activity level, TV/computer/video game usage, and smoking and alcohol use, it remains possible that these covariates did not fully account for residual potential confounding related to lifestyle. The associations between yogurt consumption and lower adiposity might also be explained by concurrent healthful dietary and lifestyle habits of yogurt consumers. Nevertheless, yogurt has been shown to augment fat loss and preserve muscle mass during weight loss in obese adults when consumed as part of an energy restricted diet [39], supporting a more direct role for yogurt in its association with obesity.

Despite greater energy and saturated fat intakes, dairy consumption was not associated with greater body weight or adiposity measures. In fact, dairy consumption was associated with smaller subscapular skinfold thickness (but not other metrics of weight or adiposity investigated in this study). Interestingly, results of dairy and calcium intake analyses were parallel, in that the highest tertile of calcium intake had smaller subscapular skinfold thickness compared to the lowest tertile. As calcium intake is strongly associated with dairy consumption, a possible explanation for the association of dairy with subscapular skinfold thickness may be due to a mechanism through which calcium is known to affect adiposity. A variety of anti-obesity effects of calcium have been described [40] including increased diet-induced-thermogenesis, increased fatty acid oxidation, increased lipolysis, decreased lipogenesis [41,42], increased fecal fat excretion [43,44], and decreased hunger or increased satiety [45], although results have been mixed [42,46,47]. Anti-obesity mechanisms of vitamin D have been proposed, including anti-inflammatory and pro-apoptotic effects in adipocytes [48,49]. Both higher yogurt and dairy consumption were associated with higher total protein intake. Dairy foods are a leading source of protein in the diet, and contribute 18% of available dietary protein in the food supply [50]. Recent research suggests that increasing dietary protein intake, including dairy protein, may be a potential strategy to influence body weight regulation and appetite control [51]. A study comparing the impact of yogurt as an afternoon snack on satiety measures in healthy women reported that a high protein (24 g) yogurt led to decreased hunger, increased fullness and lower subsequent food intake compared to a typical yogurt with 5 g of protein [52]. This suggests that increasing protein intake from dairy foods may have a beneficial impact on acute food intake which could influence chronic energy consumption and weight gain over time. 

Subscapular skinfold thickness was the only adiposity measure that consistently showed significant inverse associations among all of our primary exposure variables of interest: calcium intake, vitamin D intake, yogurt consumption, and dairy consumption. Therefore, we propose that this simple anthropometric adiposity indicator could be useful as an outcome variable in future cross-sectional and intervention studies investigating associations or effects of dairy, yogurt, vitamin D or calcium intake on regional adiposity in children. In addition, goals for healthy growth and weight management in an adolescent population are different from those in adults. The findings that the consumption of dairy foods, and related nutrients, may have a beneficial influence on an indicator of body composition during a time of rapid growth and maturation may be a practical and relevant finding than differences in body weight *per se*.

The fact that the combined 2005–2008 NHANES sample is large and representative of the U.S. population is a major strength of this study. Additionally, BMI was based on measured height and weight and reference criteria were used to assess weight status and adiposity. The analysis also controlled for potential confounders including demographic and lifestyle factors, including physical activity. This study is limited, however, by the use of the 24-hour dietary recall which may not reflect habitual intake, relies on memory, and is susceptible to over- and under-reporting. Additionally, NHANES data are cross-sectional; therefore, because the observed associations were not temporal, ultimately, causality cannot be inferred. An additional limitation is that this study did not examine all types of dairy foods, but only total dairy intake (milk, cheese, yogurt, whey) and yogurt specifically, as it has inherent differences in the texture, matrix and bacterial composition compared to other common dairy foods.

## 5. Conclusions

This study supports the role for yogurt and dairy as foods that increase intake of key shortfall nutrients in children, including potassium, calcium and vitamin D. Yogurt consumption had additional favorable associations with lower total fat and saturated fat intakes, whereas dairy was associated with higher energy intakes and higher saturated fat intakes. Despite these differences in total nutrient intakes, consumption of yogurt and other dairy foods does not appear to be associated with higher body weight or adiposity in children. In fact, consumption of yogurt and higher intake of dairy, calcium, and vitamin D intake were all independently associated with lower body fat in children as measured by subscapular skinfold thickness. Thus, these data support our hypothesis. To determine the long-term impact on weight and adiposity of inclusion of dairy products in the diets of U.S. children, intervention studies are required. It is also important to examine the mechanisms by which dairy impacts weight and adiposity in order to provide a physiological understanding.
